# The Effect of Wing-Flashing Behavior on Prey Capture Performance of San Clemente Loggerhead Shrikes

**DOI:** 10.1093/iob/obae042

**Published:** 2024-12-27

**Authors:** Y A Mora, S Sheldon, J Carrero, S M Farabaugh, D Sustaita

**Affiliations:** Department of Biological Sciences, California State University San Marcos, 333 S. Twin Oaks Valley Rd, San Marcos, CA 92096, USA; Recovery Ecology, San Diego Zoo Wildlife Alliance, 15600 San Pasqual Valley Rd, Escondido, CA 92027, USA; Recovery Ecology, San Diego Zoo Wildlife Alliance, 15600 San Pasqual Valley Rd, Escondido, CA 92027, USA; Recovery Ecology, San Diego Zoo Wildlife Alliance, 15600 San Pasqual Valley Rd, Escondido, CA 92027, USA; Department of Biological Sciences, California State University San Marcos, 333 S. Twin Oaks Valley Rd, San Marcos, CA 92096, USA

## Abstract

Loggerhead shrikes (*Lanius ludovicianus*) are medium-sized predatory songbirds that feed on arthropods and vertebrates. Prior to attacking their prey, shrikes have been observed performing “wing-flashing” behavior, consisting of rapid fluttering of the wings that seems to emphasize the white patches on their dorsal surfaces. We sought to quantify this behavior by analyzing videos of San Clemente loggerhead shrikes attacking insect and vertebrate prey, to understand whether and how wing-flashing affects prey capture performance. We measured predictors of wing-flashing behavior, wing-flashing kinematics, and prey capture performance in terms of the number of strikes required to kill prey, prey strike durations, prey escape distances, and prey survival probabilities. Juveniles were more likely to perform wing-flashing behavior than adults, and lizards elicited wing-flashing more than mice and crickets. Adult males tended to flash their wings faster than juvenile males, and although wing-flashing rates were similar between ages for females and across prey types (∼15 Hz), shrikes flashed their wings for longer durations toward lizards. Wing-flashing was generally associated with fewer strikes to kill prey and resulted in longer prey strike durations for adult shrikes, longer prey escape distances, and lower prey survival probabilities for male shrikes. Our results suggest that wing-flashing behavior of loggerhead shrikes enhances their prey capture performance, possibly by stimulating prey to move—and not to move, depending on prey type—making them more vulnerable to predatory strikes.

## Resumen

*Lanius ludovicianus* es un pájaro cantor depredador de tamaño mediano que se alimenta de artrópodos y vertebrados. Antes de atacar a su presa, han sido observados ejecutando un comportamiento de “destello alar” que muestra las alas brevemente con aleteos rápidos que parecen resaltar las manchas blancas en sus superficies alares dorsales. Intentamos cuantificar este comportamiento analizando vídeos de *Lanius ludovicianus mearnsi* atacando insectos y vertebrados, para comprender si el movimiento de las alas afecta el rendimiento de captura de presas. Medimos los predictores del comportamiento del destello alar, la cinemática del destello, y el rendimiento de captura de presas en términos del número de ataques necesarios para matarla, la duración del ataque, las distancias de escape y las probabilidades de supervivencia de la presa. Los juveniles fueron más propensos a ejecutar el destello alar que los adultos, y los lagartos provocaron este tipo de aleteo más que los ratones y los grillos. Los machos adultos tendieron a mover sus alas más rápido que los machos juveniles, y aunque las frecuencias de los movimientos de las alas fueron similares entre edades de las hembras y los tipos de presas (∼15 Hz), los pájaros aletearon durante períodos más prolongados cuando cazaban lagartos. El uso de los destellos alares generalmente se asoció con menos intentos para matar a la presa y resultó en duraciones más largas de estos ataques para los adultos, distancias de escape de las presas más largas y menores probabilidades de supervivencia de las presas para los machos. Nuestros resultados sugieren que el comportamiento de destello alar de *Lanius ludovicianus* mejora su rendimiento de captura de presas, posiblemente estimulando a las presas a moverse—o no moverse, dependiendo del tipo de presa—haciéndolas más vulnerables a los ataques depredadores.

## Introduction

Throughout the world there are various species of birds that are known to perform flashing or fluttering movements with their wings and/or tails for a variety of reasons (Daanje 1950). Among some of these taxa, these wing and tail movements have been associated with foraging behavior (Mumme et al. 2006). Flush-pursuit foraging, in which birds actively startle prey to facilitate capture, is common among small insectivorous birds (Mumme 2002). These taxa often possess contrasting patches of plumage that are displayed with their exaggerated foraging maneuvers, presumably to enhance their visual impact (Mumme 2002). Northern mockingbirds often perform wing “hitches” during foraging and in response to predators showing the underside of their wings, which allows light to pass through the white spots on the dorsal side of their wings (Sutton 1946; Hailman 1960; Selander and Hunter 1960; Horwich 1965; Burtt et al. 1994; Hayslette 2003; Dhondt and Kemink 2008; Peltier et al. 2019). European starlings (Hailman 1959), white-capped dippers (Babin 2002), and several warbler species (Jabłoński 1996, 1999; Mumme 2014) perform movements of the wings and/or tails during foraging, principally to flush visually oriented prey with their contrasting wing colorations and enhance foraging performance. In these cases, wing-flashing is thought to startle prey into motion (Sutton 1946; Hailman 1960; Jabłoński 1999; Jabłoński 2001; Babin 2002; Peltier et al. 2019), by flickering the light (Hayslette 2003; Inger et al. 2014), or by manipulating the perception of their body size (Jabłoński and Strausfeld 2000; Carlile et al. 2006). These wing and tail flashes, and the resulting sudden display of contrasting colors, play a potential role in startling prey and enhancing overall foraging performance. However, there are still some species for which wing- and/or tail-flashing movements have not been extensively studied, such as the loggerhead shrike.

Shrikes (Laniidae) are medium-sized predatory songbirds that are well known for impaling their prey on sharp objects and plants (Lefranc and Worfolk 1997; Panov 2011). Various aspects of shrike hunting behavior have been well described, and these studies have highlighted components of the predatory sequence starting with the attack (e.g., Smith 1973; Busbee 1976; Craig 1978; Sustaita et al. 2018) and ending in impaling (Wemmer 1969; Smith 1972). However, few have discussed the component acts preceding prey attacks. Like other insect-eating birds, shrikes are known to perform “wing-flashing” before attacking their prey. Miller (1931) described how in loggerhead shrikes (*Lanius ludovicianus*) “[c]age birds dance about with wings spread and tail fanned when hesitating in their attack on a large-sized animals.” Cade (1962) discussed this behavior at length in the Northern shrike (*Lanius borealis*), in six behavioral contexts: “flight-intention movements,” food-begging, courtship, hunting, agonistic interactions, and stretching. Cade (1962) suggested that wing-flashing movements were expressed most intensively during “flight intention,” which consisted of bobbing and twisting movements of the horizontally oriented body and rapid dorsoventral tail flips in concert with “wing flicks.” Smith (1973) and Busbee (1976) described variations of this behavior in their studies of loggerhead shrike predatory ontogeny. Smith reported the onset of a “spread-wing display” that occurred around 24–30 days after hatching. Busbee (1976) described a more diverse array of “fluttering” of the wings, “wing spreading” with the tail fanned, ranging from “stationary” to “flashing” of the wings half-extended during predatory encounters. To our knowledge, a video documentary on shrikes by Larry McPherson (Callaghan 2017) was the first to show wing-flashing behavior in the loggerhead shrike, but none of the aforementioned pioneering studies specifically examined the effects of wing-flashing on prey capture performance.

**Fig. 1:**
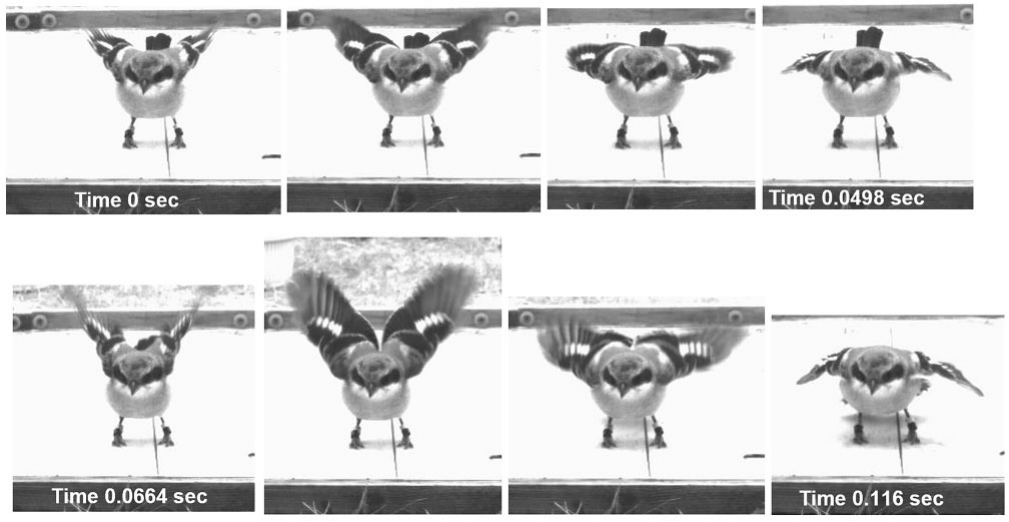
San Clemente loggerhead shrike performing two contiguous cycles of wing-flashing over the course of 0.116 s (from left to right, top to bottom).

Here we focus on characteristics of wing-flashing kinematics, and the effect that these “wing-flashing” movements have on prey capture performance in San Clemente loggerhead shrikes (*Lanius ludovicianus mearnsi*). Wing-flashing in shrikes consists of rapid, dorsoventral fluttering of the wings, with simultaneous long-axis rotations that expose the white patches on the dorsal surfaces toward the prey (Fig. 1; Supplementary Video 1). Their wings possess conspicuous white patches along their black primary flight feathers, which cover a greater extent of the primaries in males compared to females (Collister and Wicklum 1996; Sustaita et al. 2014). These wing patches seem to accentuate the behavior (Supplementary Video 2). Our objectives were to understand the context of the wing-flashing behavior and examine how it affects the shrikes’ prey capture performance, which we define as the behavioral acts and proficiency with which they attack, incapacitate, or kill prey. Based on studies of other species that perform similar behaviors (cited earlier), we hypothesized that wing-flashing should occur more frequently with prey that are unfamiliar and/or difficult to subdue (Hailman 1960). We expected that the rates (Hz) of wing movements should vary by prey class, given the differences in their “critical fusion frequencies”—the frequencies at which flickering light becomes a constant stream, which is a measure of temporal resolution of visual systems (Inger et al. 2014). For example, among the prey taxa considered here, insects show the highest critical fusion frequencies, mammals comparatively lower, and reptiles the lowest (fig. 1 of Inger et al. 2014), leading to the expectation that shrikes might adjust for these differences in temporal resolution by flashing wings fastest for insects, for example, and slowest for lizards. We also expected that wing-flashing would improve the shrike's ability to capture prey (e.g., after Mumme 2002, 2014). For example, studies have shown that wing-flashing increases insect prey escape distances to the predators’ advantage (Jabłoński 1999; Jabłoński and Strausfeld 2000). We tested these hypotheses by analyzing video sequences of prey attack behavior performed by adult and juvenile shrikes on insect and vertebrate prey types. We recorded the contexts under which wing-flashing behavior occurred, the kinematics of the “wing-flashing” movements, and the extent to which wing-flashing was associated with prey capture performance.

## Methods

### Study subjects and filming

We conducted this study at the San Diego Zoo Wildlife Alliance's San Clemente loggerhead shrike conservation breeding facility, located *in situ* on San Clemente Island, CA, USA, on six separate occasions from June 2011 to March 2024. The shrikes at this facility are wild-type birds that were hatched in the conservation breeding facility and temporarily maintained for periods ranging 0.12–7.04 years prior to their release into the wild or for breeding purposes (Munkwitz et al. 2005; Farabaugh 2013). As a result, live prey are important components of their captive diet. The shrikes are fed crickets (*Gryllus* sp.), juvenile (∼12 g) and adult (∼17 g) domestic mice (*Mus musculus*), and on occasion, wild-caught side-blotched lizards (*Uta stansburiana*) and island night lizards (*Xantusia riversiana*) (∼6 cm, ∼7 g), which are representative of the general classes of prey they encounter (and take) in the wild on San Clemente Island (Scott and Morrison 1990). In this way the shrikes attain and maintain proficiency in prey capture for their eventual release into the wild (Farabaugh 2013). Zoo staff feed the shrikes live prey daily in circular and ovular “feeding corrals” (∼0.76-m to 1.2-m diameter, 0.61-m tall; Supplementary Video 2) to promote movement of prey while preventing their escape from the enclosures.

We filmed under natural light conditions outdoors on the island, which ranged from overcast to direct sunlight depending on the weather conditions and time of day. We used frame rates of 50–250 fps and shutter speeds of one to two times the reciprocal frame rate (depending on the lighting conditions). A subset of videos featuring nine of the shrikes came from a previous study (Sustaita et al. 2018), in which a shallower square plywood-floor feeding corral (0.76-m wide × 0.30-m tall, with 0.64-cm-thick Plexiglas walls) was used in conjunction with one to two TroubleShooter HR 3GB monochrome high-speed video cameras (Fastec Imaging, San Diego, CA, USA) with Nikon AF Nikkor 24- to 120-mm f/3.5–5.6D lenses (Nikon, Melville, NY, USA) positioned at ground level inside the enclosures, approximately 1 m from one (or two orthogonal) side(s) of the corral (Sustaita et al. 2018). We recognize that the disparity in corral shapes might influence (or limit) the prey's abilities to evade their predators, so we included data from these square Plexiglas corrals only in analyses testing response variables on which corral shape should have no bearing (e.g., wing-flashing frequencies, rates, and durations). The cameras were operated remotely from a laptop computer running MiDAS OS (Xcitex, Woburn, MA, USA) from a blind outside the enclosure. In separate filming sessions conducted later, we used GoPro Hero 4, 7, and 10 cameras (GoPro, San Mateo, CA, USA) filming at 60–100 frames per second, anchored to the top of the shrikes’ regular circular and ovular hardware cloth feeding corrals. This arrangement provided dorsolateral wide-angle views of prey attacks, and longer record durations, to capture entire predatory events for another 48 individual shrikes.

In sum, we obtained prey attack footage for 45 adults (>1-year-old; range = 1.0–12.8) and 16 juveniles (<1-year-old; range = 0.12–0.90) of both sexes: 24 adult males, 21 adult females, 10 juvenile males, and 6 juvenile females (which includes data for four individuals spanning both “juvenile” and “adult” age classes). These individuals were filmed attacking 29 lizards, 40 mice, and 33 crickets for a grand total of 270 observed prey attacks. The distributions of sex, age, and prey types represented in the dataset reflect our opportunistic sampling based on which animals were available to us during the times of filming, as well as which birds actually performed for the cameras (i.e., many birds that were filmed simply did not attack prey in the time we had allotted to film them). The video recordings of feeding events were conducted in compliance with University of Connecticut Institutional Animal Care and Use Committee regulations (#E11-014), California State University San Marcos (CSUSM) IACUC protocol #17-007 and by the San Diego Zoo Wildlife Alliance IACUC exemption dated June 7, 2017.

### Data acquisition

Shrikes were chosen for filming haphazardly based on our accessibility to their enclosures, with no explicit bias. Not every individual experienced each type of prey during our study: 26 shrikes were recorded on a single prey type, 17 on two different prey types, and 14 on three different prey types. Furthermore, some individuals were recorded on the same prey type more than once, and because the study spanned multiple years, four individuals included in the analyses were coded as “juveniles” in earlier prey attack video sequences, and as “adults” in later ones. Therefore, we included “shrike identity” as a random subject effect in the mixed-effects models (e.g., Harrison et al. 2018) described later to account for nonindependence among repeated observations within subjects. For each bout of wing-flashing behavior (i.e., continuous series of wing-flashing cycles), we calculated the rates at which the wing-flashing movements were performed from the reciprocal of the number of frames to complete a single wing flap cycle (e.g., Fig. 1), averaged over multiple (1–29; mean = 6.2) flapping cycles during a given bout, multiplied by the frame rate. We calculated durations of the wing-flashing bouts as the end minus start times from the video time stamps; when durations lasted less than 1 s, we based the estimate on the number of flap cycles multiplied by the number of frames per flap, divided by the frame rate.

We conducted a survival analysis (e.g., Clark et al. 2003) to obtain individual-level prey survival probabilities as a metric of prey capture “success” (given that prey capture success in the conservation enclosures is ultimately 100% given enough time). We used R package “survival” (Turner and Nagraj 2017; Therneau 2023), which appropriately models the outcome of an event (i.e., death of prey) as a function of time, when confronted with “censored” data—cases in which the “event” never occurred during the recorded interval. “Time” for our purposes was the total attack sequence duration, from the time the attack was initiated to the time prey was immobilized or killed, subtracting periods when shrikes momentarily left feeding corrals. The survival probabilities reflected in the Kaplan–Meier plot (Kassambara et al. 2021; Fig. S1) of prey during each attack sequence were extracted from the output of the analysis and used as a response variable in a linear mixed-effects (LME) model analysis (described later). From the videos we measured aspects of prey capture performance (Table 1) that should have direct implications for predatory efficiency, in terms of time and energy invested in handling prey (after Mumme 2002; Mumme et al. 2006). These included (1) the number of strikes that resulted in killing or incapacitating prey, scored as each instance a shrike lunged at, and bit or grasped prey with its beak across video frames. (2) Prey strike duration was measured from the frame number of the initial lunge to the frame number of contact with prey (the number of frames was later converted to seconds based on the video frame rate used to film a given attack sequence). (3) Prey escape distance was estimated (visually) from images as the number of shrike body lengths between the shrike and its prey at the frame in which the prey began to flee (this was detected as an acute change in body orientation preceding their movement away from the shrike). We did not attempt to digitize actual distances because the views were often limited to single camera, nonplanar views that precluded accurate distance calibrations.

**Table 1:** Descriptions of response variables measured from 270 prey attack sequences by 57 San Clemente loggerhead shrikes

**Measurement**	**Description**
Probability of wing-flashing	The propensity of a shrike to perform wing-flashing behavior, modeled from a generalized linear mixed-effects model with “wing-flashing” or “no wing-flashing” as a binary response variable as a function of age, sex, and prey type
Flash rate (Hz)	The frequency of wing movements, calculated from the reciprocal of the average number of frames to complete a single wing-flap cycle, multiplied by the video frame rate
Flash duration (s)	The amount of time that a bout of wing-flashing was performed, calculated as the number of frames per flap multiplied by the number of flaps, divided by the video frame rate
Prey strike duration (s)	From the time the shrike initiated its strike to the time it made contact with the prey or closed its beak after missing the strike
Number of prey strikes	The number of times shrikes lunged at prey with their beaks to bite at, or grasp, prey resulting in incapacitation or death of prey
Total attack duration (s)	From the time the shrike started attacking prey to the time the prey was fully immobilized or dispatched
Prey escape distance (body lengths)	Estimated by the number of shrike body lengths (from tail tip to beak tip) between the shrike and prey at the moment prey attempted to flee
Prey survival probability	Estimated from a survival analysis of prey attack outcome (killed or censored) as a function of total attack duration

### Statistical analyses

To understand the set of conditions in which wing-flashing is performed during predatory encounters, we performed a binomial generalized linear mixed model (GLMM) using function *glmer* of R (R Core Team 2021) package “lme4” (Bates et al. 2015) to test for the effects of “prey” (crickets, lizards, and mice), age (adults *vs*. juveniles), and sex (males *vs*. females) on wing-flashing (binary response; wing-flash *vs*. no wing-flash) with shrike identity was included as a random subject effect. We performed LME models (including shrike identity as a random subject effect) using the *lmer* function in conjunction with R package “lmerTest” (Kuznetsova et al. 2017) to test the effects of prey type, sex, and age on (log_10_-transformed) wing-flashing rates and durations.

We performed the following analyses to test for the effects of wing-flashing, accounting for prey type, sex, and age, on prey capture performance: (1) a GLMM on the number of strikes required to kill or incapacitate prey, assuming a negative binomial distribution appropriate for overdispersed count data (Yirga et al. 2020), and LMEs on (2) log_10_-transformed prey strike durations, (3) log_10_-transformed prey escape distances + 0.5 (to account for “0” values when prey initiated escapes underneath the shrike), and (4) prey survival probabilities. We note for these capture performance analyses that are based on entire prey attack sequences, rather than individual bouts of prey strikes, we used the “wing-flashing” or “no wing-flashing” designation associated with the majority of prey strikes during an entire attack sequence. Furthermore, because “wing-flashing” was the predictor of primary interest, we included only the two-way interactions between wing-flashing and each covariate. Nonsignificant interaction terms were removed from models to strengthen tests of main effects. We tested for normality and homoscedasticity using normal quantile plots of residuals and plots of residuals versus fitted values of the models.

## Results

### Shrike wing-flashing behavior

Not all of the shrikes in our sample were observed to perform wing-flashing behavior; of the 57 individuals in our sample, 19 (33%) were observed to do so at some point in the videos. Furthermore, those shrikes that were observed to perform wing-flashing did not always do so prior to every attack (when multiple attacks were recorded for a given individual). When wing-flashing was performed, it typically occurred immediately upon landing close to the prey. The behavior consisted of one to several bouts of shallow dorsoventral wing-flapping strokes with the wings only partially extended (approximately one-fourth to three-fourths of full length in 80% of wing-flashing bouts), with some degree of long-axis rotation, presumably to project the white patches on the dorsal aspects of the wings more anteriorly (Fig. 1; Supplemental Video 1). Often the behavior was accompanied by a pitched (head down) body posture (i.e., “bowing”; Smith 1973) and lateral movements of the feet and body to circumscribe the prey (Supplemental Video 2).

**Fig. 2:**
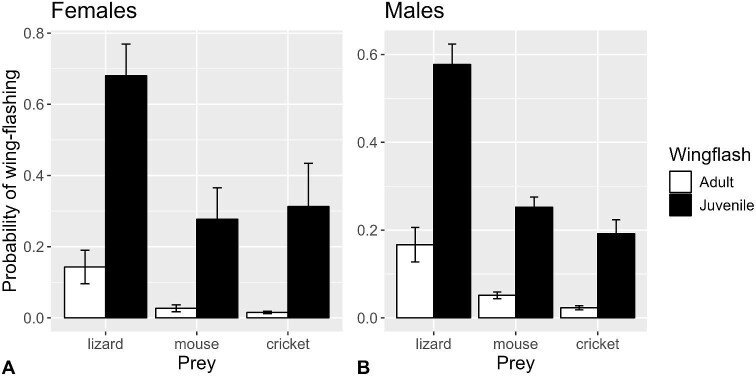
Mean (±SE) predicted probabilities of performing wing-flashing behavior for (**A**) female and (**B**) male for juvenile (filled bars) and adult (open bars) San Clemente loggerhead shrikes during prey attacks (*n* = 57 shrikes, 270 observations), from a binomial generalized linear mixed-model of the effects of shrike age, sex, and prey type on the occurrence of wing-flashing.

Overall, 41% (13/32) of males and 27% (7/26) of females, 75% (12/16) of juveniles, and 20% (9/45) of adults performed wing-flashing behavior at some point prior to attacking prey. With regard to prey type, 31% (9/29) of individuals performed wing-flashing when presented with lizards, 23% (9/40) when presented with mice, and 21% (7/33) when presented with crickets. Across all prey attack sequences, the probability of performing wing-flashing was significantly greater for juvenile shrikes than for adults (*P* = 0.0048), and for lizards compared to mice (*P* = 0.0028) and crickets (*P* = 0.0008) but did not differ between sexes (*P* = 0.3618; Table S1; Fig. 2).

**Fig. 3:**
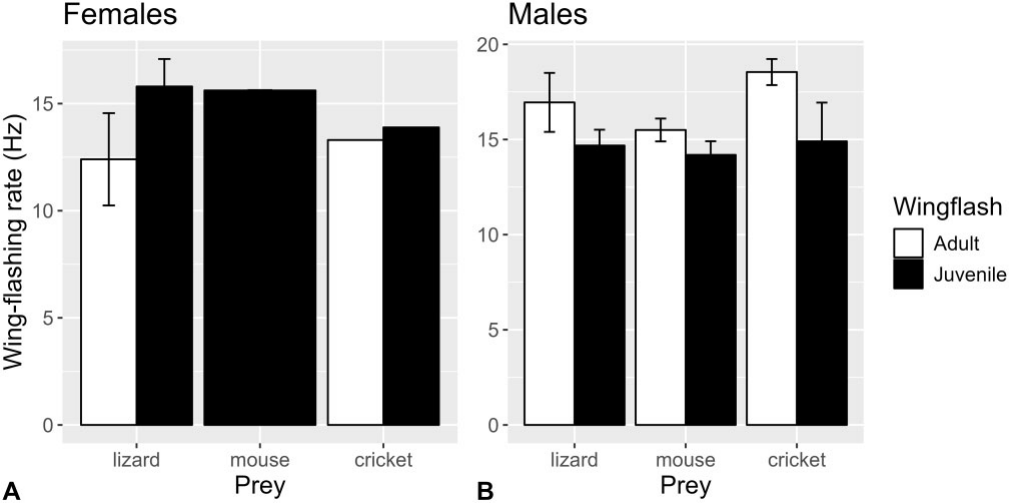
Mean (±SE) rates (Hz) of wing movements of (**A**) female and (**B**) male for juvenile (filled bars) and adult (open bars) San Clemente loggerhead shrikes while performing wing-flashing at crickets, lizards, and mice (*n* = 19 shrikes, 37 observations).

**Fig. 4:**
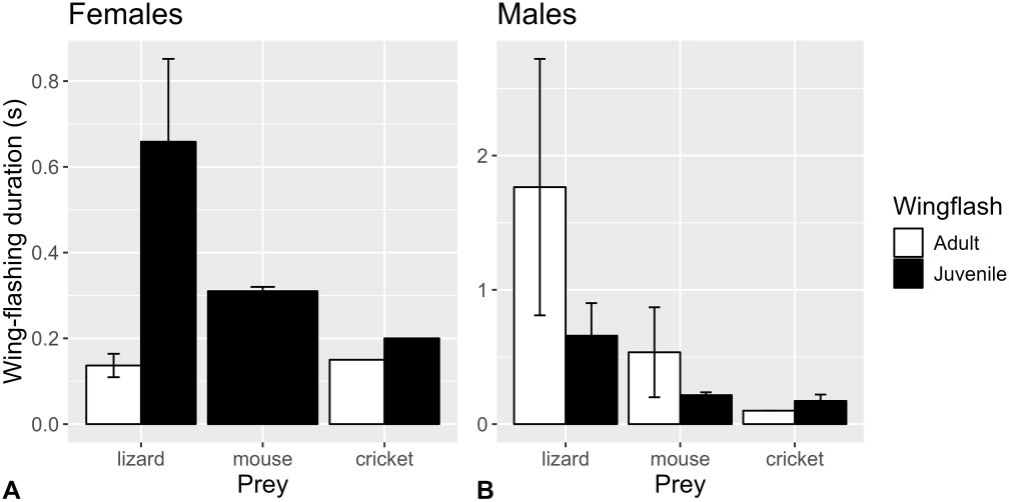
Mean (±SE) durations (s) of wing-flashing bouts of (**A**) female and (**B**) male juvenile (filled bars) and adult (open bars) San Clemente loggerhead shrikes while performing wing-flashing at crickets, lizards, and mice (*n* = 19 shrikes, 37 observations).

Shrikes moved their wings at mean ± standard deviation (*n*) rates of 15.0 ± 2.7 (37) Hz for 0.46 ± 0.55 s (37) while performing wing-flashing. There were no overall differences among prey types (*P* = 0.8167), but the difference between ages depended on sex (*P* = 0.0064): adult male shrikes showed higher wing-flashing rates than juvenile males (*P* = 0.0399), but there was no significant difference between ages for females (*P* = 0.1472; Table S2; Fig. 3), despite the fact that all 15 adult females exposed to mice did not perform wing-flashing. Incidentally, the rate at which shrikes moved their wings while performing wing-flashing behavior did not differ significantly from the rates at which they flapped their wings during ascents and descents into and out of the feeding corrals (*F*_1,43_ = 2.677, *P* = 0.1091). Wing-flashing duration differed significantly by prey type overall (*P* = 0.0096), such that lizards elicited longer wing-flashing durations than mice (*P* = 0.0442) and crickets (*P* = 0.0053; Table S3; Fig. 4). Differences between ages depended on sex (*P* = 0.0144). For females, juveniles displayed greater durations than adults (noting that adult females did not wing-flash toward mice), whereas for males the adults showed greater durations, although the effects of age were marginally nonsignificant (*P* = 0.0537 and *P* = 0.0820, respectively).

### Prey capture performance

#### Number of prey strikes

**Fig. 5:**
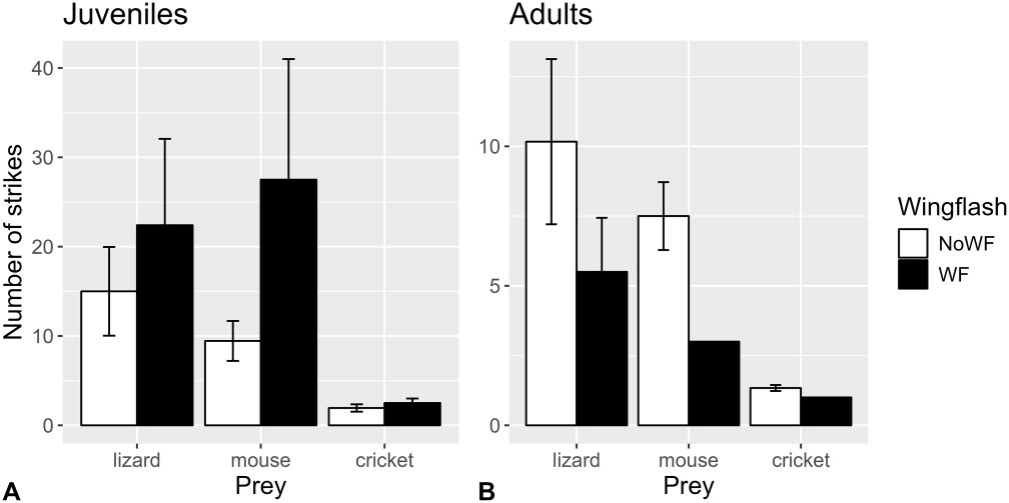
Mean (±SE) number of strikes that resulted in incapacitation or death of prey in (**A**) juvenile and (**B**) adult San Clemente loggerhead shrikes when wing-flashing did (filled bars) and did not (open bars) occur (*n* = 49 shrikes, 180 observations).

There was a significant wing-flashing × age interaction on the number of strikes required to incapacitate or kill prey (*P* = 0.0044; Table S4). We therefore separated the dataset to test juveniles and adults separately and found a significant effect of wing-flashing for juveniles (*P* = 0.0374), accounting for effects of sex (*P* = 0.3811) and prey type (*P* = 0.4304 and *P* < 0.0001 for mice and crickets; Table S4), reflecting a greater number of prey strikes associated with wing-flashing (Fig. 5). Adults, however, showed the opposite, such that wing-flashing was marginally nonsignificantly associated with a lower number of prey strikes (*P* = 0.0684), accounting for the effects of sex (*P* = 0.6527) and prey type (*P* = 0.1667 and *P* < 0.0001 for mice and crickets; Table S4).

#### Prey strike duration

**Fig. 6:**
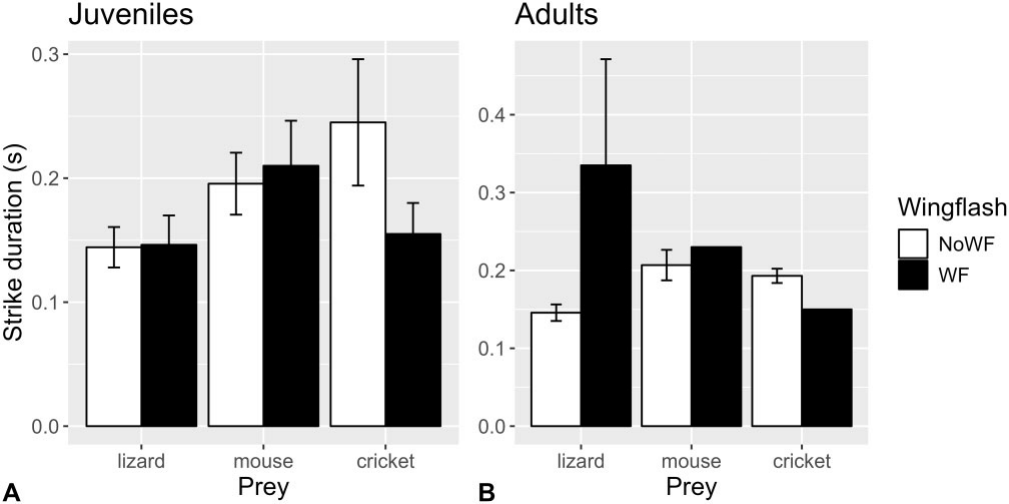
Mean (±SE) prey strike duration in (**A**) juvenile and (**B**) adult San Clemente loggerhead shrikes when wing-flashing did (filled bars) and did not (open bars) occur (*n* = 52 shrikes, 250 observations).

There was a significant wing-flashing × age interaction (*P* = 0.0454; Table S5) on log_10_-strike duration, so we performed separate tests for juveniles and adults. There was no significant effect of wing-flashing for juveniles (*P* = 0.5147; Table S5), accounting for the effects of prey type (*P* = 0.2835) and sex (*P* = 0.5332). However, prey strike durations were significantly longer for adults when wing-flashing was performed (*P* = 0.0211; Table S5; Fig. 6), accounting for the effects of prey type (*P* = 0.0096) and sex (*P* = 0.9819).

#### Prey escape distance

**Fig. 7:**
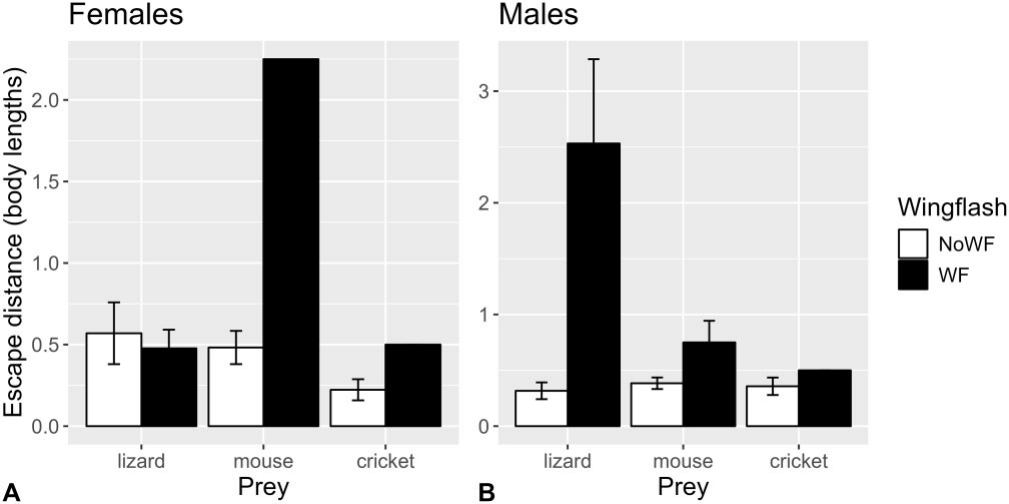
Mean (±SE) prey escape distance (body lengths) in (**A**) female and (**B**) male San Clemente loggerhead shrikes when wing-flashing did (filled bars) and did not (open bars) occur (*n* = 52 shrikes, 217 observations).

There were significant wing-flashing × prey and wing-flashing × sex interactions (*P* = 0.016 and *P* = 0.0085, respectively; Table S6; Fig. 7) on log_10_-escape distance. However, upon excluding each interaction one at a time, only the wing-flashing × sex interaction remained significant (*P* = 0.016), so we performed separate tests for females and males. For females there was no significant effect of wing-flashing accounting for the effects of age (*P* = 0.101) and prey (*P* = 0.034), after removing the nonsignificant wing-flashing × prey and wing-flashing × age interactions. For males, there was a significant effect of wing-flashing (*P* = 0.0002) accounting for the effects of age (*P* = 0.391) and prey (*P* = 0.767), after removing the nonsignificant wing-flashing × prey and wing-flashing × age interactions.

#### Prey survival probability

**Fig. 8:**
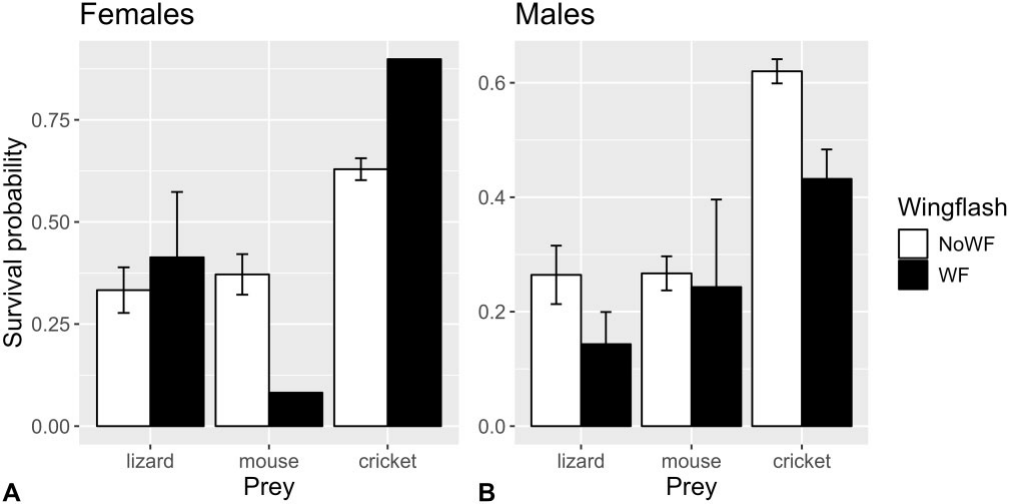
Mean (±SE) survival probability in (**A**) female and (**B**) male San Clemente loggerhead shrikes when wing-flashing did (filled bars) and did not (open bars) occur (*n* = 57 shrikes, 270 observations).

The wing-flashing × sex interaction was significant (*P* = 0.0113), resulting in separate analyses for each sex. For males, wing-flashing was associated with significantly lower prey survival probabilities (*P* = 0.0466; Table S7; Fig. 8), accounting for the effect of prey (*P* < 0.0001). For females, however, the effect of wing-flashing was not significant (*P* = 0.1096; Table S7), accounting for the effect of prey (*P* < 0.0001).

## Discussion

### Context and characteristics of wing-flashing behavior in shrikes

During encounters with live prey, 33% (19/57) of San Clemente loggerhead shrikes displayed conspicuous wing-flashing behavior, among which juveniles comprised the largest percentages. The behavior consisted of shallow dorsoventral wing-flapping strokes (Fig. 1; Supplemental Videos 1 and 2), often performed with the body pitched downward in bowing posture described by Smith (1973), such that the white patches on the dorsal surfaces of the wings projected more anteriorly. Among studies that have examined the role of wing-flashing for prey acquisition, most (if not all) seem to have focused on invertebrate prey (e.g., Jabłoński et al. 2006). Our results confirm previous findings and expand upon them by demonstrating the efficacy of wing-flashing for capturing vertebrate prey as well. Among shrikes that performed wing-flashing, lizards were most likely to elicit the behavior over mice and crickets, owing in large part to their novelty; live lizards were fed to the shrikes much less frequently. Nevertheless, we cannot exclude the possibility that something about lizards, in particular, selects for this behavior. Juveniles were generally more likely to perform wing-flashing, presumably due to their inexperience compared to adults that perhaps have learned when to perform wing-flashing toward certain prey types (Smith 1973). The fact that the most novel prey fed to the shrikes (lizards) elicited the most wing-flashing behavior from adults and juveniles alike supports this idea. Furthermore, the fact that juveniles showed greater propensities to perform wing-flashing toward ostensibly more difficult vertebrate (vs. insect) prey suggests that they might reap added benefits of wing-flashing that mitigate deficiencies in their prey capture performance; more on this next.

Despite differences in the rates of visual processing (i.e., the rate at which the world is perceived) documented by Inger et al. (2014) among insects (relatively fastest), mammals (medium), and reptiles (relatively slow), there was no significant difference in wing movement rates across prey classes, but adult males performed slightly higher wing-flashing rates than juvenile males. The Northern shrikes of Cade's (1962) study were more prone to wing-flash when confronted with prey apparently too large to kill, but wing-flashing frequencies were also similar for different kinds of prey. We did, however, find some evidence of modulation of wing-flashing behavior by prey, in terms of the differences in the durations of wing-flashing bouts, with lizards eliciting the longest durations. Perhaps the longer durations emanated from latencies in the lizards’ responses, and/or that their movements are more difficult to predict and require longer periods of assessment. Our ongoing work investigating specifically to which attributes of wing-flashing behavior prey react may shed light on this. Adult males typically performed wing-flashing bouts of higher frequencies and longer durations, possibly owing to the fact that male shrikes generally perform wing-flashing in a greater variety of contexts (Cade 1962). Among females, juveniles performed wing-flashing bouts of longer durations than adults likely due to their inexperience.

### Wing-flashing improves prey capture performance

Busbee (1976) hinted at a connection between the fluttering and bowing behaviors that Smith (1973) described of young shrikes, and their concomitant increase in invertebrate killing efficiency as they matured. Although the effect of wing-flashing on capture performance with crickets was rather diffuse, there were clearer patterns with mice, and even more so with lizards. Wing-flashing ultimately resulted in lower probabilities of survival for prey, but statistically only for male shrikes (Fig. 8). The effect of wing-flashing on prey survival was probably more equivocal for females because of the limited number of females that performed wing-flashing at crickets and mice. However, the constituent prey capture performance metrics (discussed later) showed greater differences between ages.

Wing-flashing was associated with fewer strikes required to successfully incapacitate and kill prey among adult shrikes, but a greater number of prey strikes among juvenile shrikes (Fig. 5). On the one hand this points to a potential role of wing-flashing for enhancing prey capture efficiency in adults, because this would ostensibly reduce the energetic cost of securing their prey. On the other hand, a greater number of prey strikes associated with wing-flashing in juveniles might reflect a relatively lower prey capture efficiency, or alternatively, greater number of opportunities to attack prey, with which they might otherwise have not been presented.

We found that prey strike durations were longer when wing-flashing was performed, but only for adults, and this result was primarily driven by lizards (Fig. 6). Juveniles did not seem to modulate their strikes in any way, probably a symptom of inexperience. Adults, on the other hand, might have slowed their strikes at lizards to accommodate their much more sudden and erratic movements, compared to those of relatively slower and more predicable mice and crickets with which the shrikes had more experience.

Prey escape distances were greater when wing-flashing was performed, particularly for lizards, but the effect was only significant for males. This could be an artifact of the sparse data for females, but we cannot rule out the possible influence of the greater extent of white, and presumably larger patch sizes, of males compared to females (Collister and Wicklum 1996; Sustaita et al. 2014). Previous studies have shown increased prey escape distances associated with wing-flashing and tail-fanning behaviors in other insectivorous bird species (Jabłoński 1999; Jabłoński and Strausfeld 2000; Jabłoński 2001; Galatowitsch and Mumme 2004; Jabłoński and Lee 2006). These studies have suggested that wing-flashing might accentuate the apparent sizes of the predator (Jabłoński and Strausfeld 2000), forcing insects (for example) to flee from farther distances. Others have indicated that increased escape distances benefit predators by decreasing the angular speed of the prey's image on its retina, and by decreasing the chance that the prey moves out of the bird's field of view (Jabłoński 1999). It is interesting that this pattern seems to hold for vertebrate prey as well, perhaps for similar reasons. However, the escape responses seem to emanate more from initiation of the attack, rather than the wing-flashing itself, making the effect of the wing-flashing on escape distances all the more perplexing. We predicted that prey would react to the wing-flashing behavior by moving or flinching before the shrike lunged for it (e.g., Babin 2002; Galatowitsch and Mumme 2004). Instead, we found that a larger percentage of prey did not move until the shrike actually lunged at it. We tentatively surmise that perhaps shrikes wing-flash to assess the reactivity of prey, such that their “intended” response to wing-flashing is for prey *not* to react, opting to launch their attacks when prey do not move to potentially improve their chances of capture success and economize their efforts. Hayslette (2003) proposed that the wing-flashing behavior helped mockingbirds “assess” the prey's defensive ability and whether it is worth attacking; our results are generally consistent with this idea.

This begs the question of what exactly stimulates prey to respond during wing-flashing, and this happens to be the topic of a separate parallel investigation. Cade (1962) suggested that the wing movements might serve to startle prey to move out of safety, but also serve to intimidate and distract potential enemies. Previous work on other birds (e.g., redstarts and other warblers) has suggested that insect prey react to visual stimuli, possibly aided by vibrations (Jabłoński and Lee 2006). Jabłoński and Lee (2006) found that the visual stimulus (movement of the wings and tails) from the predator is sufficient to trigger the prey escape response. Beyond birds, How et al. (2017) speculated that the “mesmerizing” wave pattern of coloration performed by cuttlefish results in an unnatural motion pattern that may cause confusion and delay the escape responses of prey. Along these lines, we cannot rule out the possibility that the “reaction” of the prey—particularly lizards—to wing-flashing might very well be to “freeze” or “fight,” rather than to “flee.” Hertz et al. (1982) found that agamid lizards tended to flee from predators at higher temperatures that maximize sprint speed, but at lower temperatures they held their ground to aggressively defend themselves. This could have substantial implications for how shrikes proceed with their attacks.

Although previous studies have documented wing-flashing in wild shrikes (Miller 1931; Cade 1962; Callaghan 2017), the extent to which San Clemente loggerhead shrikes (and other populations) perform wing-flashing during foraging in the wild is unclear. Our results nevertheless demonstrate some advantages to wing-flashing for shrike predatory performance. However, there are other plausible roles for wing-flashing in loggerhead shrikes that remain unexplored, such as during courtship, territory defense, and food-begging (e.g., Cade 1962). To this point, Smith and Ríos (2019) suggested that researchers be aware of “multiple cryptic behaviors” within wing-flashing and take into consideration the posture, movement, environmental conditions, and context during observations. Furthermore, the question of how wing-flashing evolved in shrikes remains wide open. Cade (1962) found considerable differences in the various aspects of wing-flashing behavior among behavioral contexts, ranging from “tail flipping” and “flicking” of folded wings to indicate intention to fly, to “quivering and fluttering” used by males and females during breeding to initiate courtship feeding, and finally to the “flashing” of the white wing patches used during hunting. Cade (1962) ultimately concluded that wing-flashing was likely derived from food-begging behavior, because of the similarity in the movements of the wings across these contexts to food-begging by young and courtship-feeding by adults. More detailed kinematic analyses of wing-flashing movements, such as 3D analyses of flexion, extension, and rotation of the wings, across behavioral contexts (e.g., during courtship and chick-feeding activities), may provide quantitative metrics to help assess the ontogenetic and evolutionary derivation of wing-flashing behavior in loggerhead shrikes.

## Supplementary Material

obae042_Supplemental_Files

## Data Availability

Analyses reported in this article can be reproduced using the data provided in the supplementary materials.

## Notes

Ignacio Moore (Editor-in-Chief) is a Professor of Biological Sciences at Virginia Tech. His research interests are in animal physiological ecology and behavior. He mostly studies free-living vertebrates in their natural environments and crosses traditional disciplinary lines to achieve a more complete understanding of animal function.
