# Supplementary material for: The Effect of Wing-Flashing Behavior on Prey Capture Performance of San Clemente Loggerhead Shrikes
Source: Integr Org Biol. 2024 Dec 27;7(1):obae042. doi: 10.1093/iob/obae042 (PMC11748143; doi:10.1093/iob/obae042)
Supplement: obae042_Supplemental_Files [file obae042_supplemental_files.zip › SupplementaryMaterials_Final.docx]

**
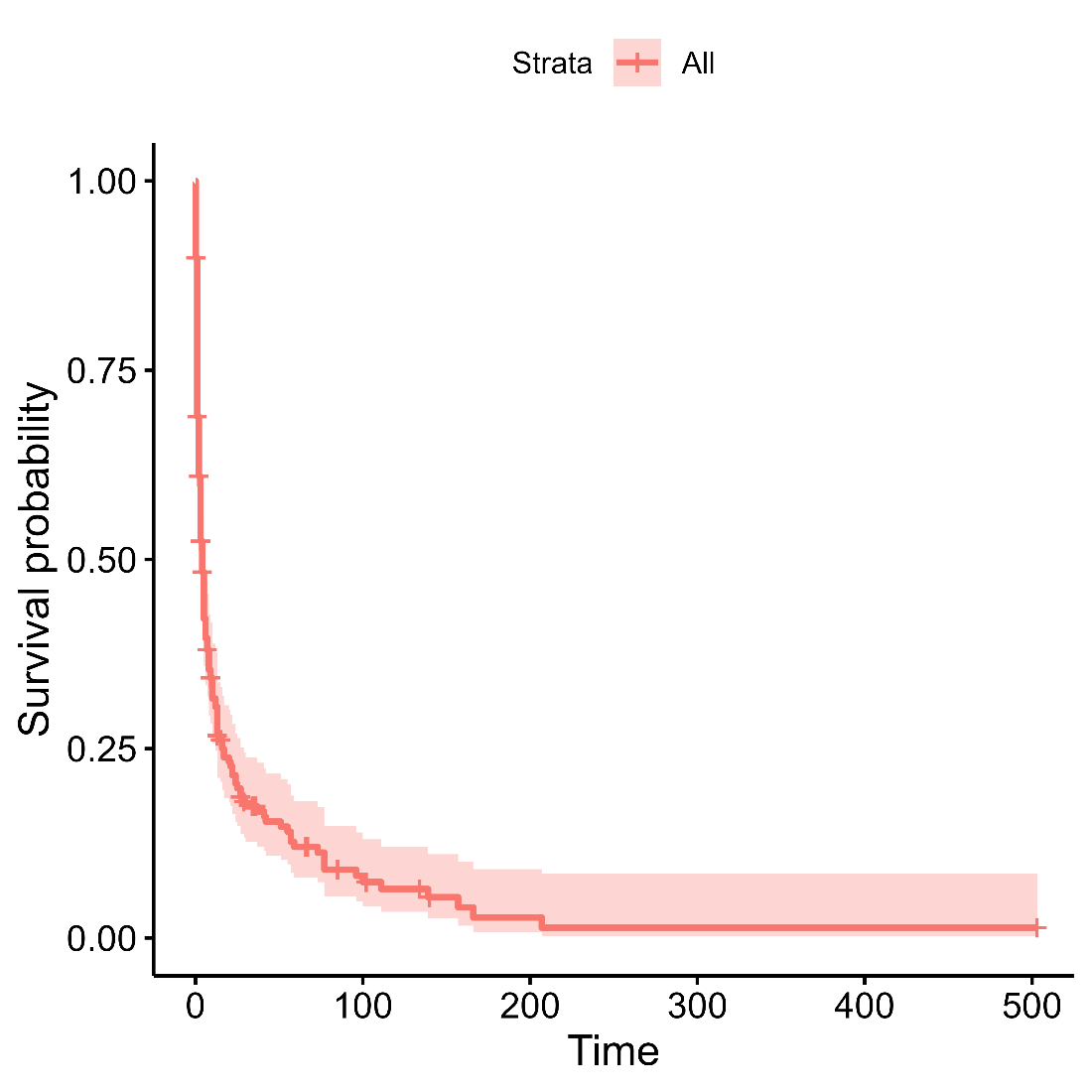
**

**Figure S1**. Kaplan-Meier plot of survival probabilities (including 95% confidence envelope) from a survival analysis to modeling the outcome of prey attack encounters (i.e., prey killed or censored) as a function of total attack sequence duration.

**Table S1.** Results of generalized linear mixed-effects models (assuming a binomial distribution) testing for the effects of age, sex, and prey type on whether or not wing-flashing was performed during the attack as a binary response, with shrike identity included as a random subject effect (*n* = 270 observations for 57 shrikes). Parameter estimates shown for full and reduced models after non-significant two- and three-way interaction terms were successively dropped (one at a time in order of decreasing *P*-value).

| Parameter |  | Value | Std. Error | *z*-value | *P*-value |
| --- | --- | --- | --- | --- | --- |
| *Full model^*^* |  |  |  |  |  |

| (Intercept) | -2.15 | 0.99 | -2.17 | 0.0300 |
| --- | --- | --- | --- | --- |
| ageJuvenile | 2.89 | 1.53 | 1.89 | 0.0594 |
| preymouse | -17.56 | 228.97 | -0.077 | 0.939 |
| preycricket | -2.88 | 1.45 | -1.99 | 0.0465 |
| sexMale | -0.053 | 1.30 | -0.041 | 0.968 |
| ageJuvenile:preymouse | 15.72 | 228.98 | 0.069 | 0.945 |
| ageJuvenile:preycricket | 0.47 | 2.26 | 0.209 | 0.834 |
| ageJuvenile:sexMale | -0.008 | 1.92 | -0.004 | 0.997 |
| preymouse:sexMale | 16.83 | 228.98 | 0.074 | 0.941 |
| preycricket:sexMale | 1.36 | 1.86 | 0.731 | 0.465 |
| ageJuvenile:preymouse:sexMale | -16.63 | 228.98 | -0.073 | 0.942 |
| ageJuvenile:preycricket:sexMale | -1.21 | 2.73 | -0.444 | 0.657 |
|  |  |  |  |  |
| *Reduced model* |  |  |  |  |
| Intercept | -2.73 | 1.03 | -2.65 | 0.0082 |
| ageJuvenile | 3.04 | 1.08 | 2.82 | 0.0048 |
| preymouse | -1.82 | 0.61 | -2.99 | 0.0028 |
| preycricket | -2.43 | 0.72 | -3.36 | 0.0008 |
| sexMale | 0.83 | 0.91 | 0.912 | 0.362 |

^*^ glmer model: wingflash ~ sex×age×prey + (1 | shrikeID), family = "binomial"; logLik = -83.2.

**Table S2.** Results of general linear mixed-effects models testing for the effects of prey, age, and sex on log_10_-transformed wing-flash rate (Hz) with shrike identity included as a random subject effect (*n* = 37 observations for 19 shrikes). Parameter estimates shown for full and reduced models after non-significant two- and three-way interaction terms were successively dropped (one at a time in order of decreasing *P*-value).

|  | Sum Sq | Mean Sq | Num DF | Den DF | F-value | P-value |
| --- | --- | --- | --- | --- | --- | --- |
| *Full model^1^* |  |  |  |  |  |  |
| prey | 0.0010 | 0.0005 | 2 | 26 | 0.077 | 0.926 |
| age | 0.0002 | 0.0002 | 1 | 26 | 0.027 | 0.872 |
| sex | 0.0147 | 0.0147 | 1 | 26 | 2.26 | 0.145 |
| prey:age | 0.0064 | 0.0032 | 2 | 26 | 0.491 | 0.618 |
| prey:sex | 0.0018 | 0.0009 | 2 | 26 | 0.136 | 0.873 |
| age:sex | 0.0228 | 0.0228 | 1 | 26 | 3.52 | 0.072 |
| prey:age:sex | 0.0006 | 0.0006 | 1 | 26 | 0.087 | 0.771 |
|  |  |  |  |  |  |  |
| *Reduced model* | |  |  |  |  |  |
| prey | 0.0023 | 0.0012 | 2 | 31 | 0.204 | 0.817 |
| age | 0.0014 | 0.0014 | 1 | 31 | 0.237 | 0.630 |
| sex | 0.0197 | 0.0197 | 1 | 31 | 3.46 | 0.073 |
| age:sex | 0.0486 | 0.0486 | 1 | 31 | 8.54 | 0.006 |
|  |  |  |  |  |  |  |
| *Males only^2^* | |  |  |  |  |  |
| prey | 0.0045 | 0.0023 | 2 | 18.72 | 0.635 | 0.541 |
| age | 0.0187 | 0.0187 | 1 | 12.58 | 5.25 | 0.040 |
|  |  |  |  |  |  |  |
| *Females only^2^* | |  |  |  |  |  |
| prey | 0.0002 | 0.0001 | 2 | 10 | 0.011 | 0.989 |
| age | 0.0217 | 0.0217 | 1 | 10 | 2.47 | 0.147 |

^1^ lmer model: log_10_(flashrate) ~ prey×sex×age + (1 | shrikeID); ^2^ lmer model reduced from: log_10_(flashrate) ~ prey×age + (1 | shrikeID).

**Table S3.** Results of general linear mixed-effects models testing for the effects of prey, age, and sex on log_10_-transformed wing-flash duration (s) with shrike identity included as a random subject effect (*n* = 37 observations for 19 shrikes). Parameter estimates shown for full and reduced models after non-significant two- and three-way interaction terms were successively dropped (one at a time in order of decreasing *P*-value).

|  | Sum Sq | Mean Sq | Num DF | Den DF | F-value | P-value |
| --- | --- | --- | --- | --- | --- | --- |
| *Full model^1^* |  |  |  |  |  |  |
| prey | 0.786 | 0.393 | 2 | 21.59 | 4.10 | 0.031 |
| age | 0.0003 | 0.0003 | 1 | 16.96 | 0.003 | 0.954 |
| sex | 0.020 | 0.020 | 1 | 13.57 | 0.209 | 0.655 |
| prey:age | 0.035 | 0.018 | 2 | 19.91 | 0.183 | 0.834 |
| prey:sex | 0.343 | 0.171 | 2 | 24.16 | 1.79 | 0.189 |
| age:sex | 0.292 | 0.292 | 1 | 19.18 | 3.05 | 0.097 |
| prey:age:sex | 0.309 | 0.309 | 1 | 16.78 | 3.22 | 0.091 |
|  |  |  |  |  |  |  |
| *Reduced model* | |  |  |  |  |  |
| prey | 1.067 | 0.533 | 2 | 29.32 | 5.47 | 0.0096 |
| age | 0.033 | 0.033 | 1 | 17.83 | 0.339 | 0.568 |
| sex | 0.128 | 0.128 | 1 | 12.39 | 1.31 | 0.274 |
| age:sex | 0.719 | 0.719 | 1 | 17.62 | 7.37 | 0.014 |
|  |  |  |  |  |  |  |
| *Males only^2^* | |  |  |  |  |  |
| prey | 1.043 | 0.522 | 2 | 18.96 | 6.70 | 0.0063 |
| age | 0.275 | 0.275 | 1 | 13.56 | 3.53 | 0.082 |
|  |  |  |  |  |  |  |
| *Females only^2^* | |  |  |  |  |  |
| prey | 0.079 | 0.040 | 2 | 10 | 0.327 | 0.729 |
| age | 0.579 | 0.579 | 1 | 10 | 4.78 | 0.054 |

^1^ lmer model: log_10_(flashduration) ~ prey×sex×age + (1 | shrikeID); ^2^ lmer model reduced from: log_10_(flashrate) ~ prey×age + (1 | shrikeID).

**Table S4.** Results of generalized linear mixed-effects models (assuming a negative binomial distribution) testing for the effects of wing-flashing (WF vs. NoWF), prey (cricket, mouse, lizard), sex (female, male), and age (juvenile, adult) on the number of prey strikes resulting in the incapacitation or death of the prey subject, with shrike identity included as a random subject effect (*n* = 180 observations for 49 shrikes). Parameter estimates shown for full and reduced models after non-significant two- and three-way interaction terms were successively dropped (one at a time in order of decreasing *P*-value).

| Parameter | Estimate | Std. Error | *z*-value | *P*-value* |
| --- | --- | --- | --- | --- |
| *Full model ^1^* | |  |  |  |
| Intercept | 2.14 | 0.181 | 11.87 | 0.0000 |
| wingflash[WF] | -0.976 | 0.453 | -2.15 | 0.0314 |
| age[juvenile] | 0.391 | 0.206 | 1.90 | 0.0576 |
| prey[mouse] | -0.314 | 0.186 | -1.69 | 0.0906 |
| prey[cricket] | -1.90 | 0.185 | -10.24 | 0.0000 |
| sex[male] | 0.013 | 0.178 | 0.08 | 0.940 |
| wingflash[WF]: age[juvenile] | 1.14 | 0.430 | 2.65 | 0.0082 |
| wingflash[WF]: prey[mouse] | 0.367 | 0.467 | 0.79 | 0.431 |
| wingflash[WF]: prey[cricket] | -0.093 | 0.656 | -0.14 | 0.887 |
| wingflash[WF]: sex[male] | 0.490 | 0.428 | 1.14 | 0.253 |
| *Reduced model* | |  |  |  |
| wingflash[WF] | 2.13 | 0.156 | 13.62 | <0.0001 |
| age[juvenile] | -0.609 | 0.340 | -1.79 | 0.074 |
| prey[mouse] | 0.394 | 0.204 | 1.93 | 0.053 |
| prey[cricket] | -0.269 | 0.170 | -1.59 | 0.112 |
| sex[male] | -1.88 | 0.174 | -10.79 | <0.0001 |
| wingflash[WF]: age[juvenile] | 1.20 | 0.421 | 2.85 | 0.004 |
| *Juveniles only*^2^ |  |  |  |  |
| Intercept | 2.73 | 0.333 | 8.20 | <0.0001 |
| prey[mouse] | -0.263 | 0.334 | -0.79 | 0.430 |
| prey[cricket] | -1.91 | 0.360 | -5.31 | <0.0001 |
| wingflash[WF] | 0.647 | 0.311 | 2.08 | 0.0374 |
| sex[male] | -0.255 | 0.291 | -0.88 | 0.381 |
| *Adults only^3^* |  |  |  |  |
| Intercept | 2.06 | 0.192 | 10.72 | <0.0001 |
| wingflash[WF] | -0.598 | 0.328 | -1.82 | 0.068 |
| prey[mouse] | -0.276 | 0.200 | -1.38 | 0.167 |
| prey[cricket] | -1.86 | 0.194 | -9.57 | <0.0001 |
| sex[male] | 0.092 | 0.205 | 0.45 | 0.653 |

^1^nb.glmer model: numstrikes ~ wingflash×age + wingflash×prey + wingflash×sex + (1 | shrikeID), family = "negative binomial"; logLik = -384.3, residual df = 168; ^2^nb.glmer model reduced from: numstrikes ~ wingflash×prey + wingflash×sex + (1 | shrikeID), family = "negative binomial"; logLik = -102.7, residual df = 28; ^3^nb.glmer model reduced from: numstrikes ~ wingflash×prey + wingflash×sex + (1 | shrikeID), family = "negative binomial"; logLik = -278.5, residual df = 132

**Table S5.** Results of general linear mixed-effects models testing for the effects of wing-flashing behavior, sex, and age on log_10_-transformed prey strike duration (s), with shrike identity included as a random subject effect (*n* = 250 observations for 52 shrikes). Parameter estimates shown for full and reduced models after non-significant two- and three-way interaction terms were successively dropped (one at a time in order of decreasing *P*-value).

|  | Sum Sq | Mean Sq | Num DF | Den DF | F-value | P-value |
| --- | --- | --- | --- | --- | --- | --- |
| *Full model^1^* |  |  |  |  |  |  |
| winglash | 0.020 | 0.020 | 1 | 222.66 | 0.686 | 0.409 |
| prey | 0.033 | 0.016 | 2 | 213.58 | 0.549 | 0.578 |
| age | 0.127 | 0.127 | 1 | 72.88 | 4.25 | 0.043 |
| sex | 0.020 | 0.020 | 1 | 82.74 | 0.661 | 0.419 |
| wingflash:prey | 0.023 | 0.012 | 2 | 210.86 | 0.394 | 0.675 |
| wingflash:age | 0.133 | 0.133 | 1 | 234.68 | 4.46 | 0.036 |
| wingflash:sex | 0.018 | 0.018 | 1 | 239.45 | 0.619 | 0.432 |
|  |  |  |  |  |  |  |
| *Reduced model* | |  |  |  |  |  |
| wingflash | 0.059 | 0.059 | 1 | 236.68 | 1.98 | 0.161 |
| age | 0.121 | 0.121 | 1 | 73.23 | 4.07 | 0.047 |
| sex | 0.005 | 0.005 | 1 | 39.90 | 0.165 | 0.687 |
| prey | 0.306 | 0.153 | 2 | 238.38 | 5.15 | 0.0065 |
| wingflash:age | 0.120 | 0.120 | 1 | 234.49 | 4.05 | 0.045 |
|  |  |  |  |  |  |  |
| *Juveniles only^2^* | |  |  |  |  |  |
| wingflash | 0.013 | 0.013 | 1 | 62.29 | 0.429 | 0.515 |
| prey | 0.081 | 0.040 | 2 | 59.81 | 1.29 | 0.283 |
| sex | 0.013 | 0.013 | 1 | 9.39 | 0.419 | 0.533 |
|  |  |  |  |  |  |  |
| *Adults only^2^* | |  |  |  |  |  |
| wingflash | 0.157 | 0.157 | 1 | 171.59 | 5.42 | 0.021 |
| prey | 0.277 | 0.138 | 2 | 175.16 | 4.77 | 0.0096 |
| sex | 0.000 | 0.000 | 1 | 30.52 | 0.001 | 0.982 |

^1^lmer model: log_10_(strikedur) ~ wingflash×age + wingflash×prey + wingflash×sex + (1 | shrikeID); ^2^lmer model reduced from: log_10_(strikedur) ~ wingflash×prey + wingflash×sex + (1 | shrikeID)

**Table S6.** Results of general linear mixed-effects models testing for the effects of wing-flashing behavior, sex, and age on log_10_-transformed prey escape distance (body lengths), with shrike identity included as a random subject effect (*n* = 213 observations for 49 shrikes). Parameter estimates shown for full and reduced models after non-significant two- and three-way interaction terms were successively dropped (one at a time in order of decreasing *P*-value).

|  | Sum Sq | Mean Sq | Num DF | Den DF | F-value | P-value |
| --- | --- | --- | --- | --- | --- | --- |
| *Full model^1^* |  |  |  |  |  |  |
| wingflash | 0.059 | 0.059 | 1 | 201.21 | 1.82 | 0.179 |
| prey | 0.253 | 0.127 | 2 | 196.16 | 3.89 | 0.022 |
| age | 0.0012 | 0.0012 | 1 | 71.06 | 0.036 | 0.850 |
| sex | 0.250 | 0.250 | 1 | 66.99 | 7.67 | 0.0072 |
| wingflash:prey | 0.275 | 0.138 | 2 | 196.49 | 4.23 | 0.016 |
| wingflash:age | 0.044 | 0.044 | 1 | 147.66 | 1.35 | 0.247 |
| wingflash:sex | 0.233 | 0.233 | 1 | 121.63 | 7.16 | 0.0085 |
|  | |  |  |  |  |  |
| *Reduced model* | |  |  |  |  |  |
| wingflash | 0.355 | 0.355 | 1 | 181.70 | 10.42 | 0.0015 |
| sex | 0.217 | 0.217 | 1 | 66.36 | 6.38 | 0.014 |
| prey | 0.118 | 0.059 | 2 | 192.00 | 1.74 | 0.179 |
| age | 0.019 | 0.019 | 1 | 43.60 | 0.572 | 0.454 |
| wingflash:sex | 0.204 | 0.204 | 1 | 140.40 | 5.98 | 0.016 |
|  | |  |  |  |  |  |
| *Females only^2^* | |  |  |  |  |  |
| wingflash | 0.045 | 0.045 | 1 | 46.51 | 1.26 | 0.267 |
| prey | 0.256 | 0.128 | 2 | 68.88 | 3.55 | 0.034 |
| age | 0.107 | 0.107 | 1 | 19.19 | 2.96 | 0.101 |
|  |  |  |  |  |  |  |
| *Males only^2^* | |  |  |  |  |  |
| wingflash | 0.470 | 0.470 | 1 | 110.76 | 14.76 | 0.0002 |
| prey | 0.0029 | 0.0029 | 1 | 21.85 | 0.090 | 0.767 |
| age | 0.060 | 0.030 | 2 | 107.96 | 0.947 | 0.391 |

^1^lmer model: log_10_(escapedist) ~ wingflash×age + wingflash×prey + wingflash×sex + (1 | shrikeID); ^2^lmer model reduced from: log_10_(escapedist) ~ wingflash×prey + wingflash×age + (1 | shrikeID)

**Table S7.** Results of general linear mixed-effects models testing for the effects of wing-flashing behavior, sex, and age on arcsine-square root-transformed prey survival probability, with shrike identity included as a random subject effect (*n* = 270 observations for 57 shrikes). Parameter estimates shown for full and reduced models after non-significant two- and three-way interaction terms were successively dropped (one at a time in order of decreasing *P*-value).

|  | Sum Sq | Mean Sq | Num DF | Den DF | F-value | P-value |
| --- | --- | --- | --- | --- | --- | --- |
| *Full model^1^* |  |  |  |  |  |  |
| winglash | 0.003 | 0.003 | 1 | 184.09 | 0.09 | 0.766 |
| age | 0.193 | 0.193 | 1 | 92.19 | 5.29 | 0.024 |
| prey | 1.097 | 0.548 | 2 | 185.47 | 15.00 | <0.0001 |
| sex | 0.219 | 0.219 | 1 | 109.42 | 6.00 | 0.016 |
| wingflash:age | 0.098 | 0.098 | 1 | 185.60 | 2.68 | 0.103 |
| wingflash:prey | 0.002 | 0.001 | 2 | 184.07 | 0.03 | 0.974 |
| wingflash:sex | 0.172 | 0.172 | 1 | 192.58 | 4.70 | 0.031 |
|  |  |  |  |  |  |  |
| *Reduced model* | |  |  |  |  |  |
| wingflash | 0.001 | 0.001 | 1 | 192.03 | 0.02 | 0.894 |
| age | 0.092 | 0.092 | 1 | 39.75 | 2.54 | 0.119 |
| prey | 4.802 | 2.40 | 2 | 198.14 | 66.43 | 0.0000 |
| sex | 0.262 | 0.262 | 1 | 109.73 | 7.24 | 0.0082 |
| wingflash:sex | 0.239 | 0.239 | 1 | 192.02 | 6.60 | 0.011 |
|  |  |  |  |  |  |  |
| *Females only^2^* | |  |  |  |  |  |
| wingflash | 0.099 | 0.099 | 1 | 82.06 | 2.62 | 0.110 |
| prey | 1.116 | 0.558 | 2 | 87.34 | 14.81 | <0.00001 |
|  |  |  |  |  |  |  |
| *Males only^2^* | |  |  |  |  |  |
| wingflash | 0.139 | 0.139 | 1 | 108.80 | 4.05 | 0.047 |
| prey | 3.818 | 1.91 | 2 | 106.96 | 55.56 | <0.0001 |

^1^lmer model: asnsqrtsurvprob ~ wingflash×age + wingflash×prey + wingflash×sex + (1 | shrikeID); ^2^lmer model reduced from: asnsqrtsurvprob ~ wingflash×prey + (1 | shrikeID)

**Supplementary Materials Video Metadata**

SupplementaryMaterials_Video1.mp4

- Bird ID: 2550
- Sex: Female
- Age: Juvenile (0.16 yrs old)
- Body Weight: N/A
- Video Clip: TSHR1_af15_cri_06-12-11
- Camera used: Fastec Troubleshooter
- Originally recorded frame rate: 250 Hz
- Current frame rate/ playback speed: 250 Hz/5 Hz (50 times slower than real time)

SupplementaryMaterials_Video2.mp4

- Bird ID: 3848
- Sex: Female
- Age: Juvenile (0.15 yrs old)
- Body Weight: 51.3
- Video Clip: GOPR0010
- Camera used: GoPro Hero 4 Session
- Originally recorded frame rate: 100 Hz
- Current frame rate/ playback speed: 60 Hz/ 60 Hz (real time)
